# Fracture de fatigue bilatérale des jambes au décours d'une marche soutenue

**DOI:** 10.11604/pamj.2014.17.10.3798

**Published:** 2014-01-14

**Authors:** Kaouther Ben Abdelghani, Faida Ajili

**Affiliations:** 1 Service de Rhumatologie, Hôpital Mongi Slim, La Marsa, Université de Tunis El Manar, Faculté de Médecine de Tunis; 2 Service de Médecine Interne, Hôpital Militaire de Tunis, Tunisie; Université de Tunis El Manar, Faculté de Médecine de Tunis

**Keywords:** Fracture de fatigue, lésions mécaniques, sport, Stress fracture, mechanical lesions, sport

## Image en medicine

La fracture de fatigue constitue 10% de l'ensemble des lésions mécaniques liées au sport. Elle est localisée dans 95% des cas aux membres inférieures et surtout au tibia (53% des cas). Son caractère bilatéral et simultané est rare et se voit particulièrement chez les sujets sportifs. Il s'agit d'un patient âgé de 36 ans, sans antécédents pathologiques notables, qui s'est présenté pour une douleur des genoux apparue simultanément, d'horaire mécanique, évoluant depuis 6 mois. Ces douleurs étaient résistantes aux antalgiques. La mobilité des genoux était conservée. Il n'y avait pas de choc rotulien. En outre, il n'y avait pas de syndrome méniscal ni d'hyperlaxité. Il n'existait pas de syndrome inflammatoire biologique. Les radiographies des genoux étaient normales. Devant le caractère persistant des douleurs, une IRM des genoux a été pratiquée. Elle a objectivé une large plage intra-osseuse en hyposignal T1 et hypersignal T2, avec un trait de fracture qui restait en hypo signal, se situant sous les plateaux tibiaux médiaux et compatible avec le diagnostic d'une fracture de fatigue bilatérale. A la reprise de l'interrogatoire, le patient a signalé que ses douleurs étaient survenues suite à une marche prolongée. Le patient a été ainsi mis sous traitement antalgique, anti-inflammatoire et avec une mise en décharge pendant trois mois. L'évolution a été marquée par une cédation partielle des douleurs. L'intérêt de ce cas est de savoir évoquer le diagnostic d'une fracture de fatigue bilatérale et simultanée, bien que rare, devant des douleurs péri-articulaires sans signes cliniques associés et avec des radiographies standard sans anomalie.

**Figure 1:**
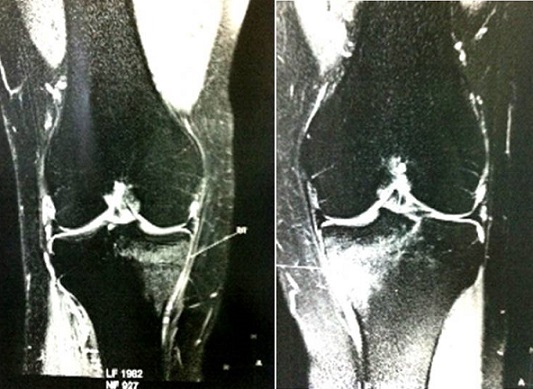
IRM des genoux en coupe frontale sur les séquences STIR : Hypersignal intra-osseux sous les plateaux tibiaux internes des 2 côtés

